# Mapping Post-Transcriptional Modifications onto Transfer Ribonucleic Acid Sequences by Liquid Chromatography Tandem Mass Spectrometry

**DOI:** 10.3390/biom7010021

**Published:** 2017-02-22

**Authors:** Robert L. Ross, Xiaoyu Cao, Patrick A. Limbach

**Affiliations:** Rieveschl Laboratories for Mass Spectrometry, Department of Chemistry, University of Cincinnati, P.O. Box 210172, Cincinnati, OH 45221-0172, USA; rossrb@ucmail.uc.edu (R.L.R.); caoxy@mail.uc.edu (X.C.)

**Keywords:** modified nucleosides, RNA sequencing, tRNA, tandem mass spectrometry, LC-MS/MS

## Abstract

Liquid chromatography, coupled with tandem mass spectrometry, has become one of the most popular methods for the analysis of post-transcriptionally modified transfer ribonucleic acids (tRNAs). Given that the information collected using this platform is entirely determined by the mass of the analyte, it has proven to be the gold standard for accurately assigning nucleobases to the sequence. For the past few decades many labs have worked to improve the analysis, contiguous to instrumentation manufacturers developing faster and more sensitive instruments. With biological discoveries relating to ribonucleic acid happening more frequently, mass spectrometry has been invaluable in helping to understand what is happening at the molecular level. Here we present a brief overview of the methods that have been developed and refined for the analysis of modified tRNAs by liquid chromatography tandem mass spectrometry.

## 1. Transfer Ribonucleic Acids

Transfer ribonucleic acids (tRNAs) are the smallest of the three main types of RNAs. They are an adapter molecule, which bridges the divide between the genetic code stored in DNA to functional proteins that are necessary for cellular viability. While a fraction of the size compared to messenger RNA (mRNA) or ribosomal RNA (rRNA), their abundance in the cell is plentiful, accounting for roughly 20% of cellular RNA content [1]. A mature, processed tRNA contains between 70–100 nucleobases and is composed of four primary domains, the D-loop, the TψC loop, the acceptor stem, and the anticodon loop. The acceptor stem carries the amino acid residue, which is covalently added to the stem by the tRNA’s cognate aminoacyl synthetase, a protein complex specific for the amino acid that is added to the individual tRNA [1]. It is the anticodon, however, which is the business end of tRNA. The anticodon complementary base pairs with the codon on an mRNA transcript, reading the genetic code for the single amino acid encoded on the transcript.

Messenger RNA decoding happens in the ribosome, a construct of rRNA and cellular proteins that make up the translational machinery. An aminoacylated tRNA enters the ribosome at the aminoacyl site via codon/anticodon recognition, and translocates to the peptidyl site where it accepts the polypeptide chain onto its amino acid to become a nascent residue within the chain. The tRNA exits the ribosome to be degraded or recharged. This decoding/translation process is key to proper protein synthesis, and incorporation of incorrect amino acid residues into the growing polypeptide chain could have devastating effects on a cell [2]. However the charging of the tRNA by its aminoacyl tRNA synthetase, recognition of a charged tRNA at the aminoacyl site of the ribosome, complementary base pairing/stability of the anticodon/codon interaction, and translocation of the tRNA at the peptidyl site are all assisted and controlled by chemical modifications to individual tRNA nucleobases, which are added post-transcriptionally.

## 2. Post-Transcriptional Chemical Modification of tRNA

Transfer RNAs contain the highest density of modifications as compared to any other class of RNA. Not only are tRNAs heavily decorated, the types of chemical covalent modifications are rich and varied in both structure and biosynthesis routes. To date, more than 100 different chemical modifications have been characterized on tRNAs from all three domains of life, and two databases, Modomics [3] and the RNA Modification Database [4], provide detailed information relating to each known modification. However, while much is known about individual modified nucleosides, far less is known about how an organism’s complement of cellular tRNAs (‘the total tRNA pool’) are modified. In fact, only a handful of organism-specific tRNA modification profiles are available [3].

**Figure 1:**
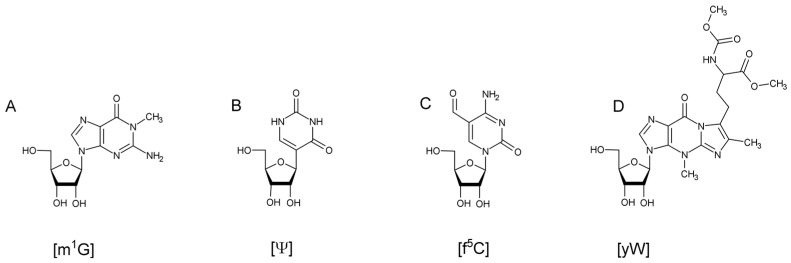
Examples of different types of RNA modifications. (**A**) 1-methylguanosine (m^1^G) methylation; (**B**) pseudouridine (ψ) isomerization; (**C**) 5-formylcytidine (f^5^C) formylation; and (**D**) wybutosine (yW) a hypermodification as the result of multiple enzymatic reactions.

Chemical modifications can include functional group addition such as methylation, formylation, glycosylation, and thiolation, or even simple isomerization [5,6,7,8,9,10]. Biosynthesis of these modifications can range from a single reaction, such as a methylation on the nucleobase or on the 2’ hydroxyl of the ribose, to ones requiring multiple enzymes and cofactors (Figure 1). Transfer RNA modifications have been found to assist in maintaining the L-shaped, tertiary structure of the tRNA [11]. They assist in tRNA recognition by its aminoacyl-tRNA synthetase [12,13] and help in stabilization and translocation of the anticodon during translation [14,15]. Despite many of these general features of tRNA modifications being known [16,17,18,19,20], for a great many individual modifications to specific tRNAs, the biological role and significance still remains unknown as there may be yet undiscovered synergistic effects among multiple modifications on the same tRNA [21] or a selective advantage for particular modifications that only arise under non-standard biological conditions [22].

To better understand the functional roles of tRNA modifications, it is important to be able to detect and place each modification within its particular tRNA sequence context. For relatively high-abundance tRNAs (e.g., 2% of total tRNA content), or for common tRNA modifications (e.g., pseudouridine), such analysis can be challenging but can be accomplished using the analytical approaches discussed below. However, for single modifications present in rare tRNAs, the analytical difficulty pushes into specialized strategies or technologies. For example, many bacteria contain the modification lysidine, which is present at the wobble base (position 34) of the anticodon on a single isoleucine decoding tRNA that is at low copy number in most cells [23]. Using *Escherichia coli* as a representative bacterium, this organism has 87 tRNA genes expressing between 42 and 45 unique tRNAs [24]. Assuming an average length for all *E. coli* tRNAs of 80 nucleotides, then lysidine exists as one modification among ~3300 bases—a needle in a haystack! The challenge of modification detection and placement becomes even more difficult within eukaryotic systems. For example, humans have over 470 tRNA genes expressing ~240 structurally unique tRNAs [25]. Generating a complete modification profile for the total tRNA pool in humans remains, at present, beyond current analytical technologies.

## 3. Analytical Approaches for tRNA Characterization

Historically, chemical modifications to tRNA were detected using paper chromatography, thin-layer chromatography, and radioisotopes [26,27]. While adequate at the time, these methods required the use of large numbers of sample and, generally, could only identify the presence of modifications outside of any tRNA sequence context. Different methods, such as reverse transcriptase polymerase chain reaction (RT-PCR) and next-generation sequencing, have been used to study tRNA modifications within the sequence context of the RNA of interest [28,29,30]. In general, however, these approaches utilize an indirect approach for modification identification—typically arising when the modification prevents amplification past the location in the tRNA sequence leading to RT stops. More recently, direct detection approaches based on next-generation technologies have been developed, usually taking advantage of a modification-specific antibody to enable purification of tRNAs from the total pool that contain the modification of interest. While it is anticipated that these approaches will continue to develop and become more widely used in tRNA modification profiling, the rest of this review will focus on the use of mass spectrometry (MS) for tRNA modification analysis.

Mass spectrometry of biomolecules was enabled by two concurrent developments of new ionization methods—matrix-assisted laser desorption/ionization (MALDI) [31] and electrospray ionization (ESI) [32]. It was in the late 1980s when researchers began to use matrix-assisted laser desorption/ionization mass spectrometry (MALDI-MS) for the analysis of large biomolecules progressing from protein analysis to the analysis of RNA oligonucleotides a few years later [33,34]. In MALDI-MS a sample is mixed with an absorbing material, the matrix, spotted onto a sample plate and allowed to dry. The sample is then subjected to a laser pulse, which ablates the sample resulting in a plume that is sampled into the mass spectrometer. While both ultraviolet (UV) and infrared (IR) wavelength lasers were investigated for oligonucleotide analysis, UV-MALDI-MS was generally more widely used due to the availability of commercial systems incorporating UV lasers as standard components [35]. MALDI-MS normally utilizes a time-of-flight (TOF) mass analyzer, which has the advantage of detecting at high mass to charge (*m*/*z*) values. A drawback with standard MALDI-TOF mass spectrometers is that these systems are not readily coupled on-line to a chromatography platform. For this reason, applications of MALDI-TOF-MS for analysis of modified tRNAs have been limited [33].

## 4. LC-MS of Oligonucleotides

**Figure 2:**
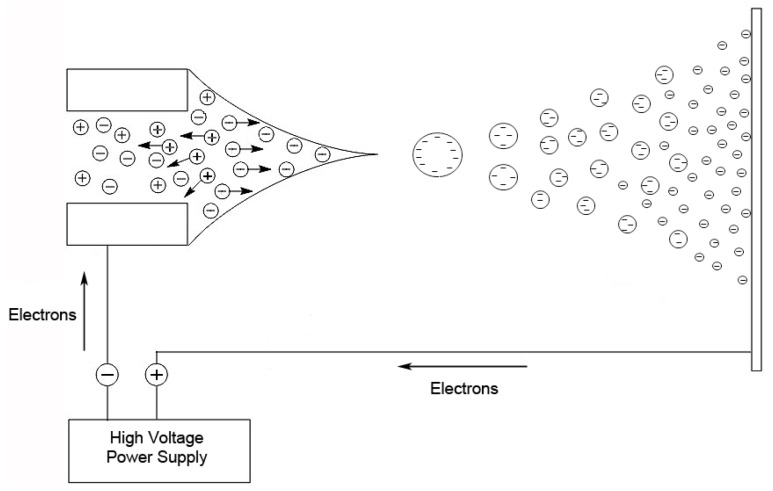
Schematic of electrospray process. Adapted from [32].

The other popular ionization method for oligonucleotides and RNA, also developed in the 1980s, is ESI [36]. In this technique, a nebulized spray is generated when a liquid is passed through a small capillary into a strong electric field [32]. The droplets in the spray are charged, and as a droplet evaporates, these surface charges get closer together until they reach the Raleigh limit [37], where columbic repulsion causes the droplet to fission into smaller droplets. This process is repeated until the ion is desorbed from the droplet or a single desolvated ion is left (Figure 2).

ESI is readily available for hyphenation to liquid chromatography platforms. High-performance liquid chromatography (HPLC) or ultra-high performance liquid chromatography (UHPLC) coupled to mass spectrometric analysis, normally represented as LC-MS, has become an extremely powerful approach for nucleic acid analysis. The phosphate backbone of RNA makes the biomolecule extremely polar as well as hydrophilic, which presents challenges for LC-MS analysis of oligonucleotides. To separate a mixture of oligonucleotides or RNAs using reverse phase chromatography, the charge on the phosphate backbone needs to be masked so that the biomolecules can be retained on the chromatographic substrate, normally some variety of silica with an 18 carbon aliphatic chain attached (C18). The addition of an alkylamine to the mobile phase, normally triethylamine (TEA) [38,39,40], accomplishes this by acting as an ion pairing reagent, masking the negative charge on the backbone. By masking the backbone, the biomolecule becomes more hydrophobic, which allows for retention on the C18 substrate. The addition of the alkylamine masks the charge and allows chromatographic retention, but to remove the alkylamine necessitates the addition of a second organic modifier into the mobile phase. The most common has been 1,1,1,3,3,3-hexafluoro-2-isopropyl alcohol (HFIP). The general consensus is that, when electrosprayed, the oligonucleotide-containing droplet rapidly evaporates due to the addition of HFIP, which has a low boiling point and high Henry’s Law constant. The rapid evaporation causes the local pH in the droplet to rise due to the presence of the alkylamine. Once the pH reaches the pK_a_ of the alkylamine, the alkylamine dissociates from the phosphate, allowing the eluted oligonucleotide to be sampled into the mass spectrometer as the free ion [39,40].

## 5. Mapping Modifications by Gas-Phase Fragmentation

More often than not, the goal in generating tRNA modification profiles by mass spectrometry is not to learn the tRNA sequence; rather. it is to identify and place modifications to specific sequence locations within that sequence. While often referred to as “sequencing by mass spectrometry”, a better phrase is “modification placement”. Regardless of the terminology used, modification placement requires one to generate sequence-specific information from the tRNA sample. Within the field of mass spectrometry, gas-phase fragmentation is the most common approach used to generate sequence-specific information. Gas-phase fragmentation is a powerful approach, as it allows one to reconstruct the underlying tRNA sequence while simultaneously denoting identities and locations of modified nucleosides within the sequence.

Gas-phase fragmentation is typically performed via two stages of mass analysis, the so-called tandem mass spectrometry (MS/MS) approach. The first stage of mass analysis is used to select the particular ion of interest for fragmentation. This selected ion is referred to as the parent or precursor ion. Onto this ion is applied some deposition of energy which results in fragmentation of the precursor into product ions which are then analyzed by in the second stage. By far the most widely used activation method for fragmentation is collision-induced dissociation (CID). CID, as its name implies, is a collision between the ion and a neutral gas atom or molecule.

**Figure 3:**
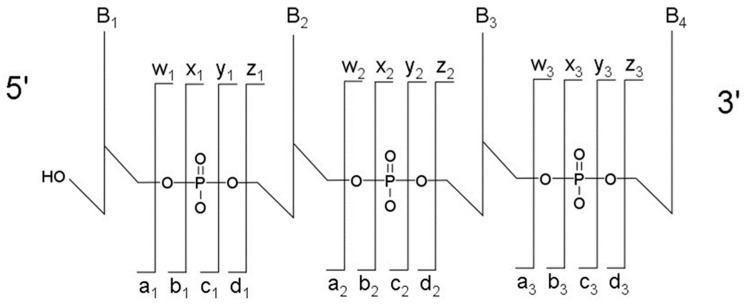
Sequence ladder of possible fragmentation sites for an RNA oligonucleotide by collision-induced dissociation fragmentation. B_1_, B_2_, etc. represent the nucleobase of the RNA, while a_1_, w_1_, etc. are the accepted nomenclature to denote the bond that is cleaved. Adapted from [41].

The most typical fragmentation pathways occur due to bond cleavage along the phosphodiester backbone or at glycosidic bonds between a nucleobase and ribose (Figure 3). CID results in the production of predominately c-type and y-type ions, using the McLuckey nomenclature [41], but other dissociation products can also be generated depending on the oligonucleotide sequence and type and amount of energy used in the collision. Upon generating the product ion mass spectrum, the underlying sequence and location of any post-transcriptional modifications can be reconstructed either manually or using software tools (described below). With either choice for reconstruction, care must be taken when assigning modifications due to side chain lability of some modifications. Some oligonucleotides containing modifications can lose all, or part, of their side chain during CID [42]. Should the most abundant fragment peak in the mass spectrum have a mass, different from the mass of any of the canonicals, or, from the loss of a terminal phosphate (−98 Th) it is probably the result of side chain loss in the modification.

## 6. The Bottom-Up Approach to RNA Modification Mapping

While mass spectrometry, in general, and LC-ESI-MS/MS, in particular, are powerful analytical platforms for mapping tRNA modifications, direct analysis of intact tRNAs is typically beyond the capabilities of common mass spectrometry instrumentation requiring specialized high-end capabilities [43]. Thus, an analytical strategy was developed by the McCloskey group, which utilizes base-specific ribonucleases (RNases) to digest tRNAs into smaller oligonucleotides (or digestion products), which are more amenable to common LC-ESI-MS/MS or MALDI-MS systems [44,45]. This approach is commonly referred to as a ‘bottom-up’ approach to modification mapping.

**Figure 4:**
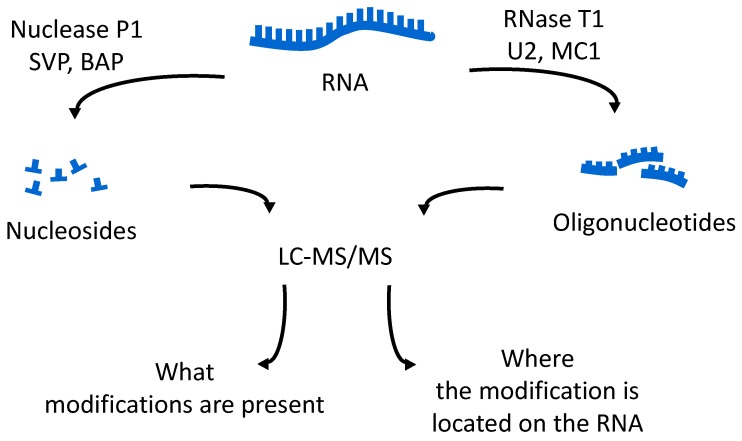
Flowchart of RNA modification mapping experiment using enzymatic digestion for generation of both nucleoside and oligonucleotide samples to be analyzed by liquid chromatography tandem mass spectrometry (LC-MS/MS). SVP: snake venom phosphodiesterase; BAP: bacterial alkaline phosphatase; RNase: ribonuclease.

The oligonucleotides resulting from the enzymatic digestion of the tRNA or tRNAs are separated and analyzed usually by liquid chromatography tandem mass spectrometry (LC-MS/MS). Because all modified nucleosides, except pseudouridine, which is an isomer of uridine, are detected at a higher mass than the canonical nucleoside, the type and position of the modification within the digestion product can be mapped using mass spectrometry. To map modifications onto an RNA sequence, two analyses are required. The first experiment is a nucleoside analysis of the system being studied. An aliquot of tRNA is enzymatically digested to its individual nucleosides [19], which can be analyzed by LC-MS/MS, or by direct infusion ESI-MS [46]. These methods allow for identification of all modified nucleosides in the sample, which will then be mapped back onto the oligonucleotide. The second analysis is the bottom-up characterization of the tRNA, using LC-MS/MS to map the position of modifications onto the digestion product (Figure 4). Using the bottom-up approach necessitates multiple overlap between the generated oligonucleotides. This is accomplished by the use of multiple base-specific RNases.

## 7. Base-Specific Nucleases for Modification Mapping

Under ideal conditions the RNA researcher would have a base specific RNase for each of the four canonical bases enabling a four-lane approach to modification mapping similar to that used in Sanger sequencing methods for DNA. Unfortunately, only two base-specific enzymes are available commercially, RNase T1 and RNase A. RNase T1 cleaves at unmodified guanosine residues and *N*^2^-methylguanosine, resulting in a digestion product containing a 5’ hydroxyl and a 3’ phosphate. RNase A cleaves at pyrimidine residues and certain modified pyrimidines, also generating a 5’ hydroxyl and a 3’ phosphate. Given the higher specificity of RNase T1, this RNase is often the first enzyme of choice for bottom-up RNA modification mapping, with RNase A often supplementing information obtained from RNase T1 (see below).

A third base specific ribonuclease, RNase MC1, has recently been described for RNA modification mapping by mass spectrometry [47]. MC1 has been found to cleave at the 5′ terminus of uridines and pseudouridine. While not yet fully exploited, this RNase offers several advantages to modification mapping strategies. Firstly, this enzyme should be attractive for tRNAs from organisms having a high GC content in their genome, as the uridine-specificity should result in longer digestion products yielding higher mapping coverage. Secondly, as cytidine and uridine differ by only 1 Da, digestion products containing a mixture of C’s and U’s can be challenging to map accurately by LC-MS/MS unless specific transitions are previously identified [48]. MC1 should generate digestion products with a known number of uridines, thus defining all remaining pyrimidines within the digestion product as cytidine.

## 8. Modification Mapping of Total tRNA Pools—Total tRNA Modification Profiles

### 8.1. Quantitative Nucleoside Profiles

One of the easiest approaches for examining tRNA modifications is to simply focus on the total nucleoside census, obtained by digestion of the pool of tRNAs by non-specific nucleases and phosphodiesterases (Figure 4). A variety of reports have established the appropriate analytical methods and techniques required to obtain quantitative information via HPLC or LC-MS platforms [18,49,50,51,52]. Dedon and co-workers have used nucleoside profiles, in combination with other analytical measurements, to examine how tRNA pools vary during the stress response for specific bacteria [22,53]. Helm and colleagues have shown that these new quantitative LC-MS approaches can reveal low abundance modifications in samples presumed to be devoid of such signals [54]. While nucleoside-based analyses are quite powerful, these measurements alone cannot reveal site-specific changes to individual tRNA modification profiles.

### 8.2. tRNA Purification and Serial Analysis

Moving beyond nucleoside profiles, historically, mass spectrometric mapping of modifications is performed on a single RNA at a time. Most of the published literature on tRNA modification mapping is focused on single tRNAs, which have been extracted from the total tRNA pool. Single tRNA probing was recently used by Hori and co-workers to map the modification 7-methylguanosine (m^7^G) to position 49 on tRNA^Leu^ from *Thermoplasma acidophilum*, a novel position for this particular modification [55]. Suzuki and Suzuki established the gold standard in this arena by developing chaplet chromatography for isolation of individual tRNAs from a more complex pool [56]. Using this approach, they purified 22 individual tRNAs from mammalian mitochondria and mapped modifications onto seven previously uncharacterized tRNAs [57].

### 8.3. Multiple RNases with Total tRNA Pools

**Figure 5:**
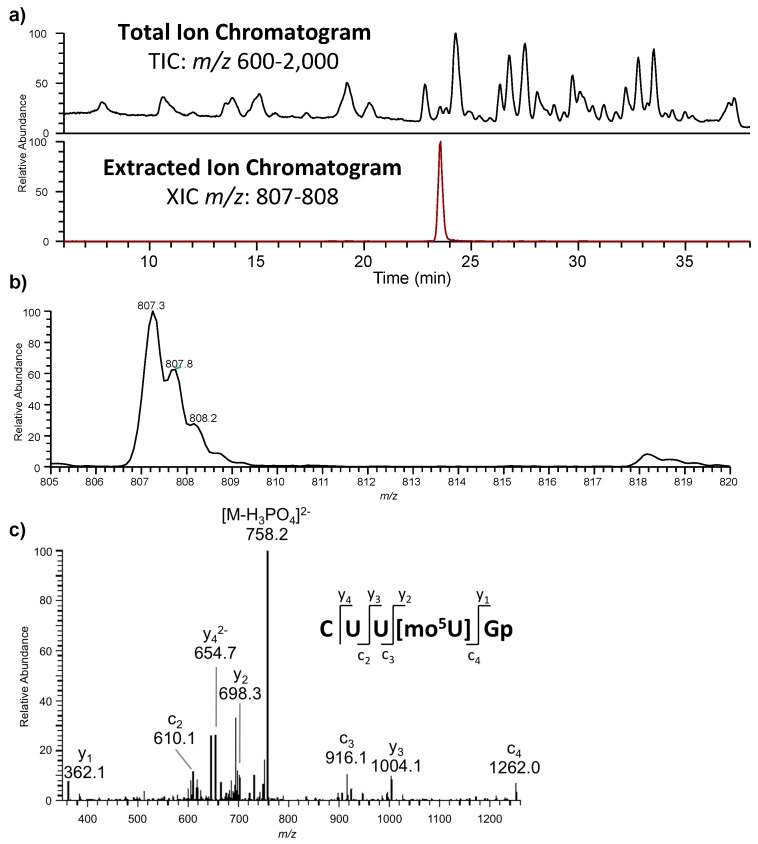
Representative example of RNA modification mapping by LC-MS/MS. (**a**) Total ion chromatogram (TIC) and extracted ion chromatogram (XIC) for RNase T1 digest of *Lactococcus lactis*. Only one peak for the XIC of *m*/*z* 807 is detected; (**b**) This doubly-charged *m*/*z* value is consistent with a digestion product of composition (U_3_CGp + 30). Two known modified nucleosides have a mass change of 30 Da: 5-methoxyuridine (mo^5^U) and 5-methyl-2-thiouridine (m^5^s^2^U). As only the former was found in the nucleoside digest, mo^5^U is most likely the modified nucleoside in this digestion product; (**c**) Tandem mass spectrometry (MS/MS) data confirming sequence as CUU[mo^5^U]Gp, which maps onto Ala-tRNA^UGC^. Figure adapted from [58] with permission.

An alternative to individual isolation of tRNAs is the development of approaches for tRNA modification mapping directly from total tRNA pools [58]. A number of analytical and technical challenges exist when moving beyond single tRNA analyses. For example, with *E. coli* total tRNAs, RNase digestion will result in hundreds of digestion products of various length, each of which must be separated and analyzed by LC-MS/MS to obtain complete coverage of the tRNA sequence modifications. A common strategy used to improve such sequence coverage is the use of multiple RNases, which generate complementary digestion products that can improve modification mapping accuracy. An example is shown in a recent publication where the modification profile for the total tRNA pool from the organism *Lactococcus lactis* was determined by LC-MS/MS [58]. In that example, three separate enzymatic digestions were performed to increase the information content enabling the placement of 18 modified nucleosides onto the 40 tRNA sequences for this organism (Figure 5).

### 8.4. The Exclusion List Approach

While relatively brute-force approaches using multiple RNases can be used to generate total tRNA modification profiles, our lab has focused on alternative analytical strategies wherein the goal is to increase information content while reducing the overall amount of data generated by the bottom-up characterization of total tRNA pools. As noted earlier, understanding that the goal of RNA modification mapping is only to identify and locate modified nucleosides within the parent tRNA sequence, one can take advantage of existing tRNA sequence information to improve LC-MS/MS information content.

One such approach is through the use of an exclusion list during LC-MS/MS [59,60,61]. An exclusion list in LC-MS/MS is a list of oligonucleotide *m*/*z* values that should not be selected for CID-MS/MS. Understanding that, with the exception of pseudouridine, modifications result in an increase in the mass of the digestion product, one can generate a list of *m*/*z* values that correspond to the tRNA sequence (or sequences) in the absence of modifications. Such information is readily obtained through tRNA gene or organism-based genomic information. By excluding these unmodified digestion products from MS/MS analysis, only digestion products containing post-transcriptional modifications will be analyzed thereby increasing the information obtained during an LC-MS/MS experiment. This approach was demonstrated to yield greater coverage of the total tRNA pool from *E. coli* than standard bottom-up approaches (Table 1) and was used to map tRNA modifications for a previously uncharacterized organism, *Streptomyces griseus*.

### 8.5. The CARD Approach

**Figure 6:**
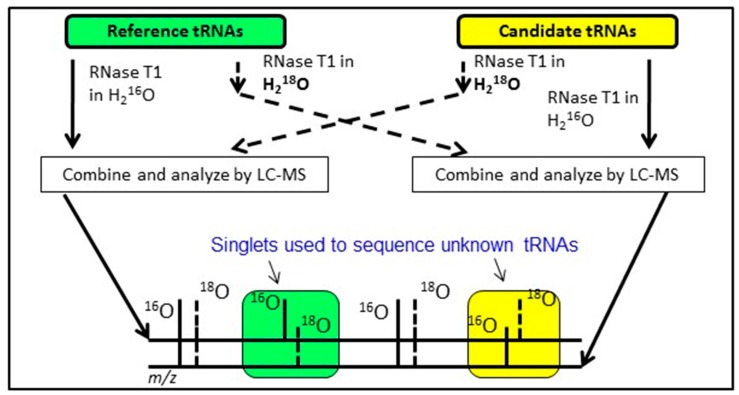
Schematic outline of comparative sequencing of total transfer ribonucleic acids (tRNAs) from an unknown (candidate) organism using a known (reference) organism by isotope-labeling and LC-MS. tRNA endonuclease digestion products that are equivalent between organisms will appear as doublets (separated by 2 Da) in the mass spectral data; digestion products that are different between the two organisms will appear as a singlet. Figure adapted from [67] with permission.

An alternative approach having similar goals as the exclusion list strategy is to conduct bottom-up analysis of two tRNA mixtures, where the modification profile of one tRNA sample is known and the modification profile of the second tRNA sample is unknown. By differentially labeling the RNase digestion products from the two samples using various heavy isotope strategies [62,63,64,65], digestion products that are equivalent between the two samples will appear as doublets in the LC-MS/MS data. Missing doublets (i.e., singlets) occur when the underlying tRNA sequences differ between the two samples or when the two samples are modified differently (Figure 6). Regardless, this Comparative Analysis of RNA Digests (CARD) [66,67,68] approach allows one to more quickly identify changes in tRNA modification profiles, such as between wild-type and mutant strains, or can be used to generate the total tRNA modification profile from a previously uncharacterized organism.

## 9. Tools to Analyze MS Data

Bottom-up modification mapping experiments can generate a significant amount of LC-MS/MS data, even when strategies, such as an exclusion list or CARD are used. Manual analysis and annotation of such data would render moot any advantages of characterizing multiple tRNAs within the same experiment. Thus, like in the field of proteomics, another prime area of research and development is into new software tools that can reduce the time necessary to process large LC-MS/MS datasets. One of the original software tools, Simple Oligonucleotide Sequencer (SOS), was written to annotate oligonucleotide MS/MS data [69]. While SOS was the first user-friendly tool for ab initio oligonucleotide sequencing, it did not automate the process of data annotation. Additional tools soon followed, including RNA Mass Mapping (RRM) [70], Ariadne [71], and RoboOligo [72].

RRM was developed primarily for MALDI-MS data to enable RNA identification by matching RNase T1 digestion products to those predicted from RNA sequences available in a database. Ariadne uses a probability-based algorithm to match possible modified oligonucleotides against a database of genomic sequences. RoboOligo is a more modern, automated version of SOS allowing for ab initio reconstruction of MS/MS data. While each of these programs significantly improves data analysis and annotation, additional tools are needed to keep up with the advances in mass spectrometry technology and approaches.

## 10. Conclusions

Modification mapping of tRNAs has moved from a cumbersome, sample- and time-intensive endeavor into an era where modification profiles for total tRNA pools are now feasible. More promising, recent advances in chromatography and mass spectrometry technology have made interrogation of complex mixtures of tRNAs even easier. Chromatography improvements are pushing towards the goal of baseline separation of nearly every RNase digestion product in the sample that much closer to reality. At the same time, developments in mass spectrometry technology have allowed for improved detection limits and sensitivity, bringing the detection of low-abundance RNA modifications within the grasp of the field. Anticipated future advances [30,73,74], including an even greater synergy between genomic-based sequencing or direct RNA-sequencing technologies and mass spectrometry wherein sites previously identified by mass spectrometry can be interrogated in a high throughput fashion [75], suggest tRNA modification mapping will become a routine analytical method impacting a variety of biological studies.

## Figures and Tables

**Table 1:** Number of RNase T1 digestion products identified (and percentage out of 73 predicted) from *Escherichia coli* total tRNA via replicate runs of standard Data Dependent Acquisition-based LC-MS/MS and LC-MS/MS using an exclusion list. Adapted from [59] with permission.

Method	Replicate 1	Replicate 2	Replicate 3
Standard DDA	51 (70%)	50 (68%)	51 (70%)
Exclusion list	59 (81%)	60 (82%)	60 (82%)
% increase	16%	20%	18%
